# Investigating Optimum Conditions for Developing Pozzolanic Ashes from Organic Wastes as Cement Replacing Materials

**DOI:** 10.3390/ma15062320

**Published:** 2022-03-21

**Authors:** Suhail Zaffar, Aneel Kumar, Naeem Aziz Memon, Rabinder Kumar, Abdullah Saand

**Affiliations:** 1 Department of Civil Engineering, Faculty of Architecture & Civil Engineering, Mehran University of Engineering and Technology, Jamshoro 76062, Pakistan; naeem.aziz@faculty.muet.edu.pk; 2 Department of Civil Engineering, Faculty of Engineering, Quaid-e-Awam University of Engineering, Science & Technology, Nawabshah 67480, Pakistan; abdullah.saand@gmail.com

**Keywords:** rice husk ash, wheat straw ash, cow dung ash, strength activity index, weight loss, calcination, XRD

## Abstract

This research was performed to investigate the optimum conditions for developing pozzolanic ashes from organic wastes to be used as cement replacement materials. The organic wastes explored in the research are rice husk ash (RHA), wheat straw ash (WSA), and cow dung (CDA). When the organic waste is turned into ash, it develops a pozzolanic character due to the presence of silica. However, the presence of reactive silica and its pozzolanic reactivity depends on the calcination temperature, duration, and grinding. In this research, the organic wastes were calcined at three different calcination temperatures (300 °C, 400 °C, and 800 °C) for 2, 4, 6, and 8 h duration. The obtained ashes were ground for 30 min and replaced by 20% with cement. The samples containing ashes were tested for compressive strength, X-ray diffractometry (XRD), weight loss, and strength activity index (SAI). It was observed that the RHA calcinated at 600 °C for 2 h showed better strength. However, in the case of WSA and CDA, the most favorable calcination condition in terms of strength development was obtained at 600 °C for 6 h duration. The highest SAI was achieved for the mortar samples containing CDA calcinated at 600 °C for 6 h duration (CDA600-6H). The other two ashes (RHA and WSA) did not qualify as pozzolan according to the ASTM C618 classification. This was due to the presence of silica in crystalline form and lower surface area of the ash material. In this research, the ash was ground only for 30 min after calcination which did not contribute to an increase in the specific surface area and thus the pozzolanic activity. The materials ground for the higher duration are recommended for higher SAI.

## 1. Introduction

Concrete is the most consumable material in construction and its demand is continuously increasing [1]. The main ingredient required in the production of mortar or concrete is cement. The production of cement clinker is responsible for the release of 8–10% of CO_2_ in the environment [2,3]. Overall, the construction industry is reported to be responsible for CO_2_ emissions greater than 20% in the environment [4]. Since the production of cement is an energy-intensive process, the substitution of supplementary cementitious material (SCM) becomes a promising way to produce alternative binding material for the construction industry [4,5]. In addition, the disposal of untreated waste materials from industries and agricultural lands in open-air landfills has always been an environmental concern [6]. Further, the availability of land for dumping waste is becoming limited with time. This leads to the discharge of waste materials into rivers and lakes or burnt in the open air which causes air pollution as well as health hazards [2]. Utilizing such waste materials which are rich in silica extends the range of building materials to be used as cement’s substitute and can solve the ecological problems [6,7].

In past, several studies have been conducted to investigate the use of industrial by-products and agricultural wastes as a partial alternative to cement [5,8,9]. The most used industrial by-products as an alternative to cement are reported to be fly ash, slag, and silica fume [10,11]. In addition, different agricultural wastes in the form of ash are also widely used in concrete as cement replacement material (CRM) [12,13,14]. Generally, the common practice for the disposal of agricultural wastes is open-air burning [2]. This open-air burning process not only emits harmful gases but also fades cementitious properties which restrict its use as CRM in the production of cementitious material [7]. In this regard, incineration of agro-wastes in a controlled condition is a promising method to reduce environmental pollution and retain the material properties.

Sahan and Aydin [10] reported the highest strength, up to 10% substitution of RHA with cement. Chandra et al. [6] also observed the highest strength development rate at 10% replacement. Overall, the optimum replacement of RHA is selected in the range of 10–20% [4]. The enhanced strength is due to the availability of amorphous silica which increases pozzolanic performance [15,16,17]. Other than RHA, many researchers have also utilized WSA as partial cement replacing material and reported enhanced properties [18,19]. The overall properties of WSA modified concrete depend upon calcination temperature and fineness of the material [19]. In the study of Birick et al. [20], wheat straw was calcinated at 570 °C and 670 °C for 5 h, and good quality WSA was obtained at 670 °C calcination. Memon et al. [21] investigated the behavior of WSA at four different calcination temperatures ranging from 500 °C to 800 °C with an interval of 100 °C change in temperature. The calcination duration was fixed at two hours. They reported the optimum burning temperature at 600 °C for the better pozzolanic performance. Ataie et al. [22] reported the presence of higher amorphous silica content at 600 °C calcination for 1 h.

In recent years, the utilization of CDA in concrete has also been explored due to the presence of pozzolanic properties [23]. Sirri et al. [24] observed an increase in strength by utilizing CDA up to 15%. Ramachandran et al. [23] also reported better strength and higher durability by utilizing CDA as cement’s substitute. Many studies agree on the optimum calcination temperature of 600 °C to produce WSA and RHA with better pozzolanic behavior. However, the effect of calcination duration has not yet been explored and remains the topic of discussion. In addition, to the authors’ best knowledge, limited studies are reported on CDA to be used as a potential substitute to cement. Furthermore, the calcination temperature and the calcination duration for CDA containing highly reactive silica are still novel and yet to be explored. In this regard, this research focuses on investigating the optimum calcination temperature and calcination duration for developing highly reactive pozzolanic ashes from organic wastes as cement replacing materials.

## 2. Methodology

### 2.1. Materials

Type I ordinary Portland cement (OPC) conforming to ASTM C150 [25] specifications was utilized in the research. The cement was obtained from a locally available materials supplier (Lucky Cement, Karachi, Sindh, Pakistan) in the vicinity of Jamshoro. Natural hill sand passing from the #30 sieve and retained at the #60 sieve was utilized for the preparation of mortar samples. Rice husk was acquired from nearby rice mills, located in the vicinity of Jamshoro district. Wheat straw was obtained from wheat-growing areas located in the vicinity of Hyderabad, Pakistan. The maximum fiber length of wheat straw was 25 mm. Cow dung was collected from cow farms located in the Jamshoro region. It was collected in dry form and crushed into a smaller size to obtain uniform calcination.

### 2.2. Calcination of Ashes

All the organic wastes (rice husk, wheat straw, and cow dung) used in the research were subjected to heat treatment, to convert into ashes. The heating was given in an electric muffle furnace (POL-EKO-SU32, Wodzislaw Slaski, Poland). To obtain rice husk ash (RHA), wheat straw ash (WSA), and cow dung ash (CDA), the heating was performed at 300 °C, 600 °C, and 800 °C for 2, 4, 6, and 8 h duration. After heating the material at the required temperature and duration the sample was allowed to cool in the open air. All the obtained ashes were ground in the ball mill for 30 min and sieved from the #200 sieve before use as CRM. The chemical compositions of all organic ashes are shown in Table 1.

### 2.3. Sample Preparation

The mortar samples of size 50 mm^3^ were prepared by substituting 20% of the activated ashes by weight of the cement. The sample was prepared using a cement to sand ratio of 1:2.75. The water-to-binder ratio was kept constant at 0.49. The mortar mix design ratio and replacement level were selected following (ASTM C 311) [26]. The details of the mortar mix are presented in Table 2. The nomenclature for mixes is adopted in such a way that it signifies the type of ash used in a mortar mix. For example, the control mix denotes a mix containing only cement without ashes. RHA300-2H, RHA300-4H, RHA300-6H, and RHA300-8H signify rice husk ash calcinated at 300 °C for 2, 4, 6, and 8 h. The mixing, compaction, and molding of mortar specimens were performed following ASTM C305 [27] and ASTM C109 [28] guidelines. The samples were demolded after 24 h and left for 7 days of water curing.

### 2.4. Testing

Mineralogical analysis on RHA, WSA, and CDA samples calcined at 300, 600, and 800 °C was performed by X-ray diffractometry testing instrument (XRD, JDX-3532, Jeol, Tokyo, Japan).

The compressive strength of the 50 mm mortar cube at 7 days of moist curing was determined following ASTM C109-21 standard [28]. The pozzolanic activity of the ash samples at different calcination temperatures was examined as per the ASTMC618 specification [29].

The strength activity index (SAI) was determined on cubes at 7 days of curing by replacing 20% cement with a weight of the ash. SAI was then calculated as per ASTM C311 [26] using the following formula: SAI = (A/B) × 100, where A = average compressive strength of ash blended cubes, MPa (psi), and B = average compressive strength of control mix cubes.

## 3. Results and Discussion

### 3.1. X-ray Diffraction

#### 3.1.1. X-ray Diffraction Analysis of RHA at Different Duration and Calcination Temperatures

**Figure 1:**
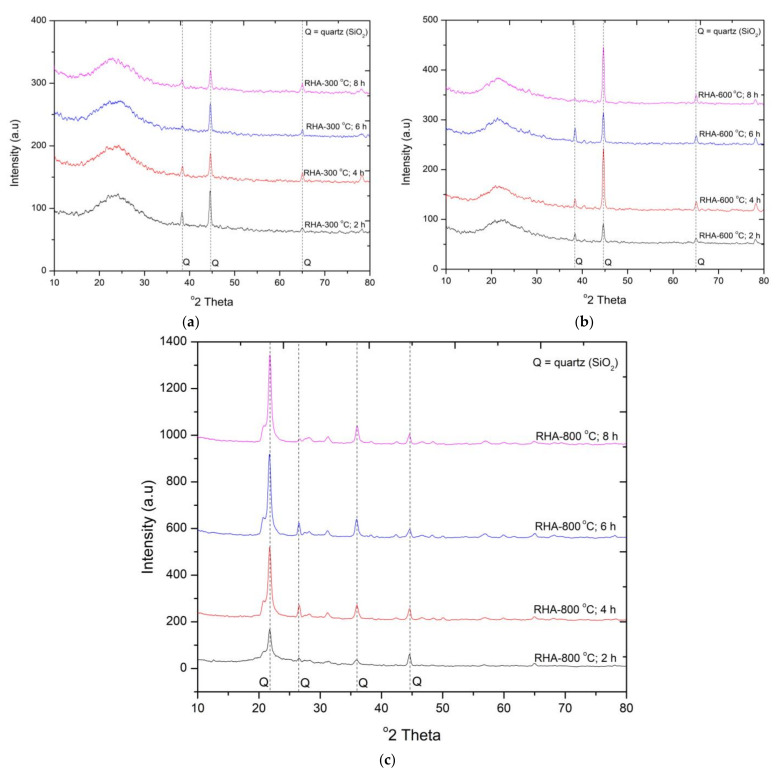
XRD pattern of rice RHA calcinated at (**a**) 300 °C (**b**) 600 °C and (**c**) 800 °C.

Highly reactive RHA contains a large amount of amorphous silica with the presence of silanol groups [30]. Combustion temperature and time are important factors in determining whether silica remains amorphous or becomes crystalline in RHA. The XRD analyses were performed to identify the changes in the development of amorphous and crystalline phases at different combustion temperatures (300, 600, and 800 °C) and durations (2, 4, 6, and 8 h). The XRD patterns of RHA depicting the crystallographic structure of silica are shown in Figure 1. The intense broad hump observed for the RHA samples at 300 °C indicated that silica remained essentially amorphous at 2θ = 22° with partial traces of crystalline quartz phase of silica reflected at 2θ =38 °, 44° and 65°, respectively. Incineration at 300 °C showed the amorphous silica with mild peaks of crystalline quartz. The intensity of peaks at 600 °C and 800 °C remained unchanged at 4, 6, and 8 h of incineration. Small amounts of amorphous SiO_2_ are present after calcination at 600 °C, and almost no amorphous silica remains after the treatment at 800 °C. This shows that the presence of silica in crystalline form occurs at higher temperatures. This is also evident from Figure 1b,c; the intensity of the peaks at 600 °C and 800 °C remains sharp at all calcination durations. The results are also consistent with the finding reported in Patel’s research [30].

#### 3.1.2. X-ray Diffraction Analysis of WSA at Different Duration and Calcination Temperatures

**Figure 2:**
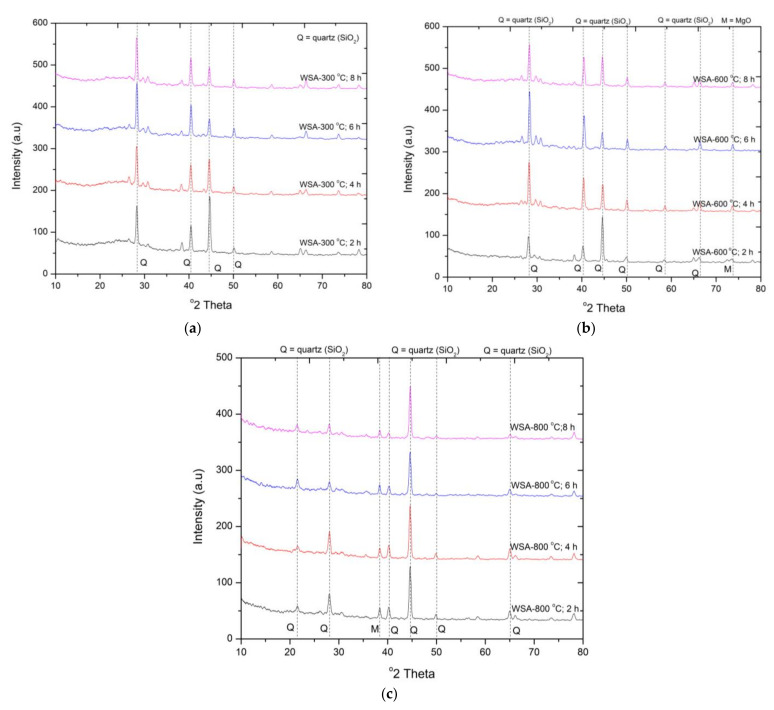
XRD pattern of rice WSA calcinated at (**a**) 300 °C (**b**) 600 °C and (**c**) 800 °C.

The mineralogical phases of silica in WSA at different combustion temperatures and duration are presented in Figure 2. The intense sharp peaks for the WSA samples at 300, 600, and 800 °C show the crystalline reflections of silica at 2θ = 28°, 45°^,^ and 45°. Overall, the crystalline structure of silica remains unchanged at the calcination temperature of 300 °C and 600 °C at all durations (2–8 h). However, the peak intensity is relatively higher at 2θ = 45° for 300 °C calcination for two hours. A similar pattern was observed for calcination temperature 600 °C at all burning durations (Figure 2b). This is in contrast with the study reported by Memon et al. [21]. In their study, the amorphous phase of silica in WSA was obtained at 600 °C. In our research, the major transformation of crystalline silica into amorphous form was observed at 800 °C calcination temperature with the sharp peak of crystalline silica at 2θ = 45°. Some mild peaks were also transformed into amorphous form during the calcination process for longer durations. This shows that the reactive silica is present in WSA at 800 °C calcination for 8 h.

#### 3.1.3. X-ray Diffraction Analysis of CDA at Different Duration and Calcination Temperatures

**Figure 3:**
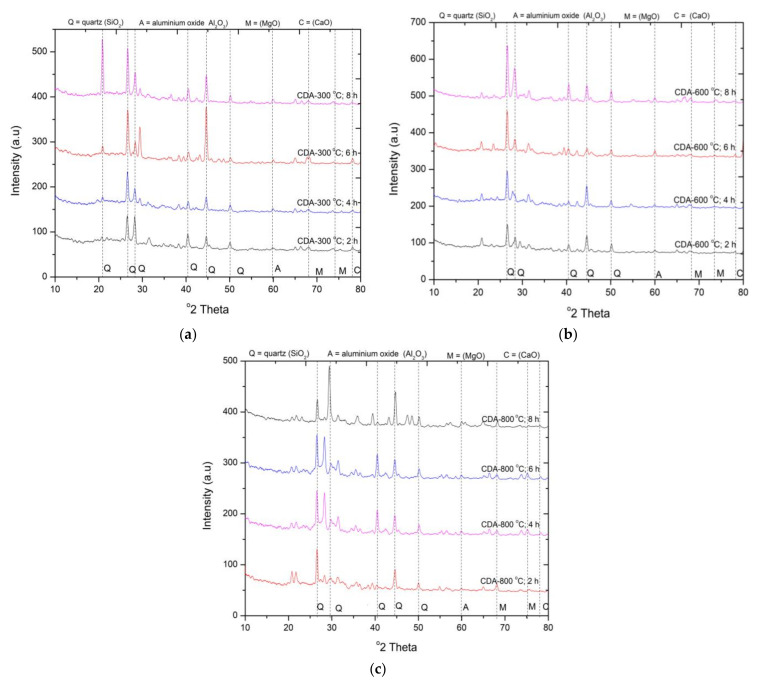
XRD pattern of rice CDA calcinated at (**a**) 300 °C (**b**) 600 °C and (**c**) 800 °C.

The XRD pattern of CDA at different calcination temperatures and duration is shown in Figure 3. Figure 3a shows the calcination of CDA at 300 °C for 2, 4, 6, and 8 h. It can be concluded from the obtained XRD spectra of CDA that silica is the major phase followed by traces of Al_2_O_3_, MgO, and CaO. The sharp peaks of crystalline quartz were identified at 2θ = 26° and 45°. The increase in the burning duration increases the crystallinity in the material. The formation of a new crystalline quartz peak was observed at 2θ = 21.6° at 300 °C calcination for 8 h. Sharp peaks at 2θ = 22°, 26°, and 45° were identified, which shows the presence of crystalline silica. Besides mild peaks of Al_2_O_3_ at 2θ = 60 °, MgO at 2θ = 68° and 74° and CaO at 2θ = 78° were also observed in the obtained spectra of CDA. Similarly, calcination at 800 °C at different duration also showed the presence of strong peaks of crystalline silica in the CDA. This shows that at the higher temperature the material is transformed into a crystalline phase from the amorphous phase. The findings are consistent with Patel et al. [31]. Based on the XRD patterns shown in Figure 1a–c, the amorphous silica in CDA is identified at the 600 °C calcination temperature for 6 h.

### 3.2. Compressive Strength and Strength Activity Index (SAI)

#### 3.2.1. Compressive Strength of RHA

**Figure 4:**
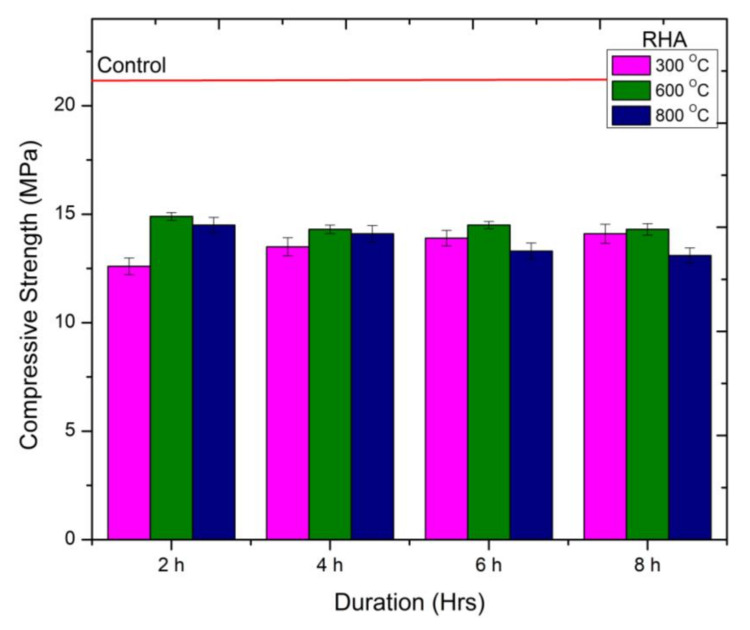
Compressive Strength behavior of RHA-cement mortar at different calcination temperatures.

The behavior of compressive strength of mortar samples containing RHA at different calcination temperatures and duration is present in Figure 4. It can be observed that the compressive strength of mortar samples containing RHA treated at higher durations during 300 °C calcination temperature is increasing. The increase in strength was observed as 7%, 9%, and 11% compared to RHA300-2H. However, a significant drop in strength was observed compared to the control mix containing cement. The drop in strength was between 32 and 40%. A lower drop in strength was recorded when RHA was treated for higher durations. The highest strength gain was recorded for the RHA sample treated at 600 °C for 2 h (RHA600-2H). The increase in strength is due to an increase in pozzolanic activity of RHA-cement mortar [32,33]. The improved pozzolanic performance is attributed to the presence of high reactive silica present in a material (Figure 1b). The strength for the remaining mixes calcinated at this temperature for a higher duration was observed to be identical but 4% and 31% lower than the mix (RHA600-2H) and control mix, respectively. the utilization of an RHA calcined at 800 °C for a higher duration caused reduction in compressive strength up to 12% compared to that sampled containing RHA calcined at 600 °C. While a drop of 38% in compressive strength was recorded compared to control mix. The decline in compressive strength is attributed to the poor pozzolanic activity due to the formation of crystalline silica at 800 °C calcination. This can be inferred from the XRD graph shown in Figure 1c. from the experimental results, it can be concluded that the high reactive RHA can be achieved at 600 °C calcination temperature for two hours. The obtained results are also in lined with Xu et al. [8].

#### 3.2.2. Compressive Strength of WSA

**Figure 5:**
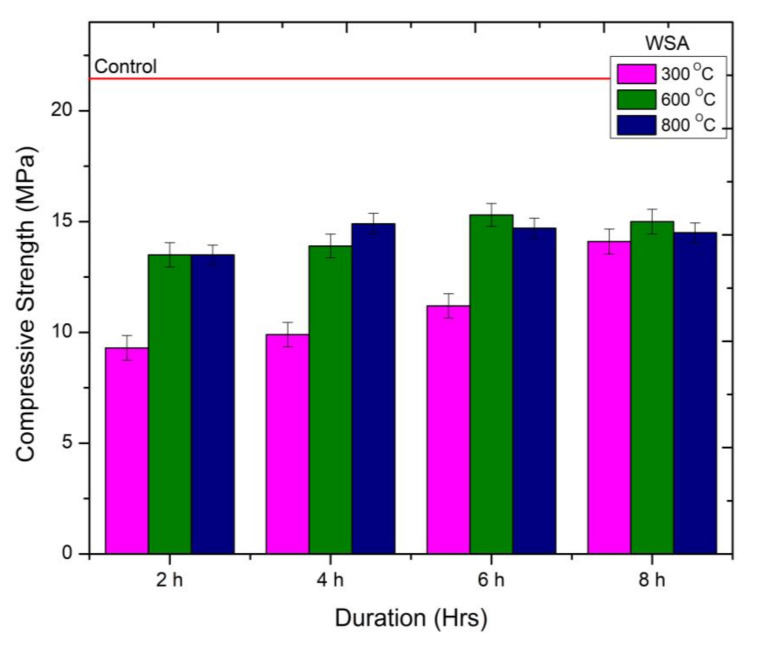
Compressive Strength behavior of WSA-cement mortar at different calcination temperatures.

The behavior of compressive strength of mortar samples containing WSA at different calcination temperatures and duration is presented in Figure 5. It can be observed that the compressive strength of mortar samples containing WSA treated at higher durations during 300 °C calcination temperature is continuously increasing. The increase in strength was observed as 6%, 17%, and 34% compared to WSA300-2H. However, a significant drop in strength was observed compared to the control mix containing cement. The drop in strength was between 45 and 68%. This loss is also significantly higher than the RHA samples. The highest strength gain was recorded for the WSA sample treated at 600 °C for 6 h (WSA600-6H) compared to the rest of the calcination temperature and durations. The improved pozzolanic performance is attributed to the presence of an amorphous form of silica in higher proportions (Figure 2b,c). However, for this mix, a significant drop of 26% in compressive strength was recorded compared to the control mix. The utilization of WSA calcined at 800 °C for a higher duration caused a reduction in compressive strength up to 11% compared to samples containing WSA calcined at 600 °C for 6 h. However, the mortar samples containing WSA treated 800 °C for 4 h also showed nearly a similar magnitude for strength as WSA600-6H. The decline in compressive strength is attributed to the poor pozzolanic activity due to the presence of crystalline silica. This can be referred from the XRD graph shown in Figure 2a–c. The compressive strength obtained in this study for mortar samples containing WSA600-2H is nearly identical with the study of Memon et al. [21]. In their study, they only considered the calcination duration for 2H. from the experimental results it can be concluded that the optimum calcination temperature of 600 °C is required to obtain high reactive WSA when calcinated for 6 h (WSA600-6H).

#### 3.2.3. Compressive Strength of CDA

**Figure 6:**
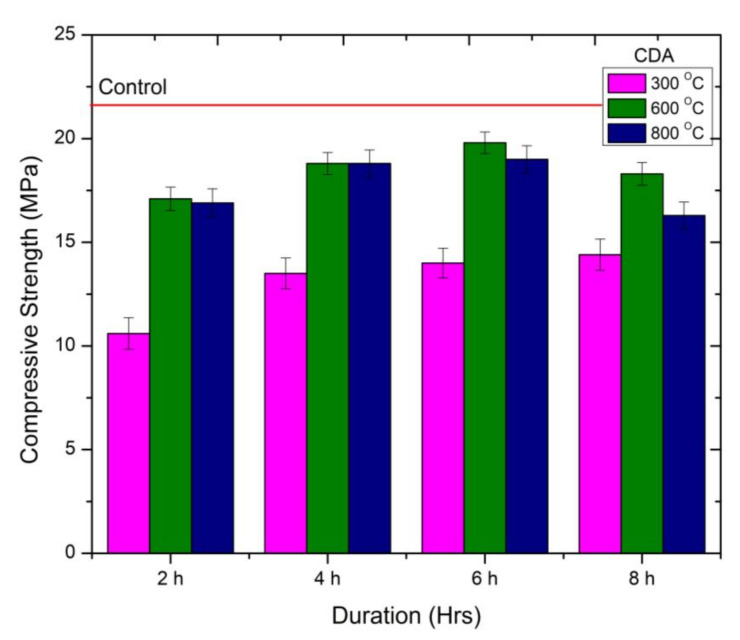
Compressive Strength behavior of CDA-cement mortar at different calcination temperatures.

It can be observed from Figure 6 that the compressive strength of mortar samples containing WSA treated at higher durations during 300 °C calcination temperature is continuously increasing. The mortar samples containing 600 °C calcinated CDA showed better strength development compared to other temperatures and duration. At this temperature, the strength gain was recorded for up to 6 h of calcination duration. The increase in strength was observed as 10%, and 15% compared to CDA600-2H. Further calcination caused a drop in strength up to 8% compared to mix CDA600-6H. The decline in strength was due to the phase transformation of silica elements present in the material. CDA calcinated at 600 °C for 6 h depicted the highest compressive strength compared to other calcination temperatures at different durations. The strength was recorded as 19.8 MPa, which is 5% lower than the control mix.

CDA calcinated at 600 °C and 800 °C for 4 h duration presented the identical compressive strength. Compared to RHA and WSA samples, the performance of CDA as cement replacement material in terms of compressive strength was exceptional. The significant rise in strength was due to higher pozzolanic reactivity, which is attributed to the presence of highly reactive silica. This is also evident from the XRD pattern shown in Figure 3b. Calcination of CDA at higher temperatures for higher duration results in lower compressive strength. The decline in compressive strength is attributed to the formation of crystalline silica at higher temperatures. It can be concluded that the optimum calcination temperature of 600 °C is required to obtain high reactive CDA when calcinated for 6 h.

#### 3.2.4. Strength Activity Index (SAI)

**Figure 7:**
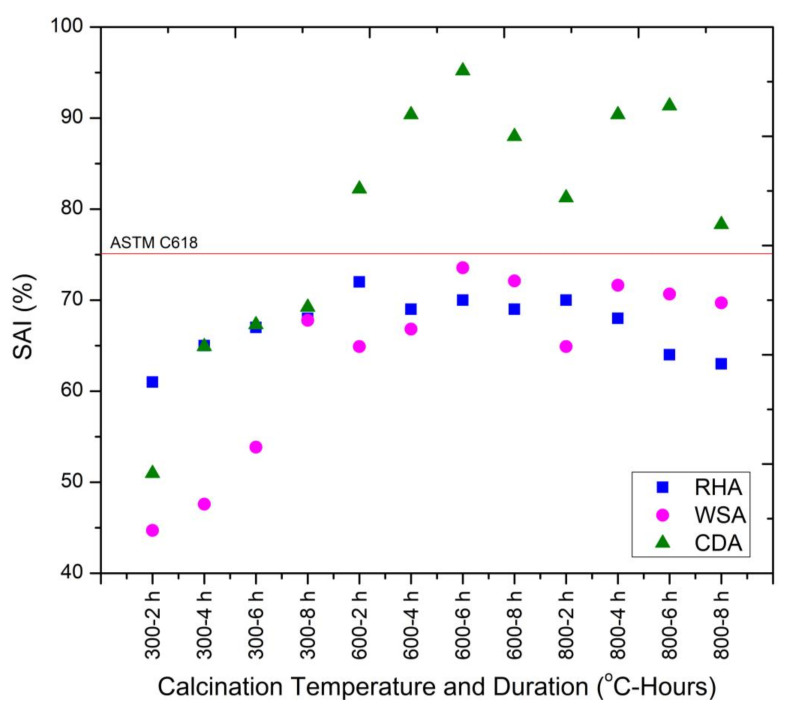
SAI of RHA, WSA, and CDA samples at different incineration temperatures.

Strength activity index (SAI) or pozzolanic activity index (PAI) is one of the most important tests that measures the compressive strength development rate of mixtures containing pozzolans. Figure 7 shows the SAI of RHA, WSA, and CDA calcined at different temperatures for different durations when used as a 20% substitute by weight of the cement. According to ASTM C618, the material can classify as pozzolan when its SAI is equal to or greater than 75%. This means that the mortar mix containing pozzolan should have the compressive strength equal to or greater than 75% of the control mix at 7 or 28 days. It can be noted that all the mixes containing CDA calcinated at 600 °C and 800 °C for 2, 4, 6, and 8 h qualified as pozzolanic material. The highest SAI was achieved for the mortar sample containing CDA calcinated at 600 °C for 6 h duration (CDA600-6H). This was due to the presence of reactive silica in the amorphous form. It is also noted that none of the ashes (RHA and WSA) except CDA qualified as pozzolanic material. The reason is attributed to the presence of crystalline phases of silica which, showed a considerable reduction in the SAI. Another reason for lower SAI is the fineness of the material. According to Kiattikomol et al. [34], fineness has a significant influence on SAI. In this research, the calcinated ashes were ground for only 30 min, which did not result in the required pozzolanic activity index as per ASTM C618. Memon et al. evaluated the effect of the specific surface area of WSA on the SAI and reported higher SAI corresponding to the higher surface area. They also reported inadequate SAI for WSA when ground for 30 min. Cordeiro et al. [35] evaluated the effect of the specific surface area of RHA on the PAI and reported higher PAI corresponding to the higher specific surface area.

### 3.3. Weight Loss

**Figure 8:**
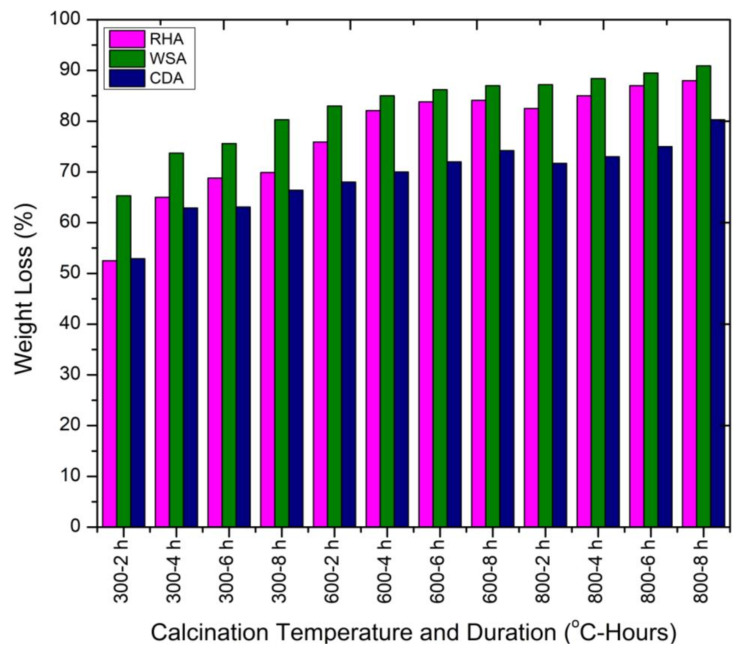
Weight loss of RHA, WSA, and CDA samples at different incineration temperatures.

Weight loss is an important parameter in terms of obtained mass to be used as a substitute for cement after calcination. The weight losses of RHA, WSA, and CDA subjected to different calcination temperatures and durations are shown in Figure 8. The increase in weight loss was observed with the increase in calcination temperature. The highest weight losses—85, 91%, and 78%—were obtained during two hours at 800 °C calcination temperature for RHA, WSA, and CDA, respectively. The WSA content obtained during calcination for two hours at this temperature was in line with the study of Memon et al. and Xu et al. [8,21]. The weight loss was due to the decomposition of organic matter present in the agricultural wastes into carbon [20].

## 4. Conclusions

This research was conducted to evaluate the optimum conditions for obtaining different ashes that can be used as a partial substitute for cement. In this research, three different ashes (RHA, WSA, and CDA) were considered as potential partial cement replacing materials. The prime focus was to find the optimum calcination temperature and duration to obtain highly reactive RHA, WSA, and CDA to be used in cement-based materials. The ashes were prepared at controlled burning temperatures of 300 °C, 600 °C, and 800 °C for 2, 4, 6, and 8 h duration. Based on the experimental results, the following findings are drawn.

- Based on the XRD patterns the silica was presented in the amorphous state when all ashes (RHA, WSA, and CDA) were calcinated at 600 °C. The optimum calcination duration for producing good quality CDA and WSA was recorded as 6 h, while the duration for obtaining highly reactive RHA was evaluated as 2 h.
- CDA samples presented considerable strength development compared to RHA and WSA samples. The highest compressive strength was obtained for the mix (CDA600-6H) containing calcinated CDA at 600 °C for 6 h. The strength was slightly lower (3%) than the control mix. However, the reduction in strength was considerably greater in the case of mortar mixes containing RHA and WSA as cement replacement material.
- The highest SAI was achieved for the mortar samples containing CDA calcinated at 600 °C for 6 h duration (CDA600-6H). RHA and WSA did not qualify as pozzolan according to ASTM C618 pozzolan classification. Along with the presence of crystalline silica, another major reason was the lower specific surface area of the RHA and WSA. In this research, the material was ground for only 30 min after calcination, which did not contribute to the significant pozzolanic activity. The materials ground for the higher duration are recommended for a higher strength activity index.
- An increase in weight loss was observed with the increase in calcination temperature. The obtained content of CDA, WSA, and RHA after calcination at evaluated optimum temperature (600 °C) was calculated as 28%, 15%, and 25%, respectively.

## 5. Recommendation

The current study focused on investigating the optimum conditions for developing pozzolanic ashes from organic wastes as cement replacement materials. The production of cement is an energy intensive process and emits a significant amount of CO_2_ into the environment. The partial substitution of cement with organic wastes turns out to be a promising way to reduce the carbon footprint. Generally, the organic wastes are turned into ashes under open-air burning. The open-air burning process not only emits harmful gases but also fades cementitious properties which restrict its use as CRM in the production of cementitious material. In this regard, the calcination in controlled conditions is a promising method to reduce environmental pollution and retain the material properties compared to open-air burning. In this research the optimum calcination conditions were obtained to develop pozzolanic ashes containing reactive silica. Since the production of ash in a controlled condition is also an energy intensive method and releases CO_2_ into the environment, the authors, based on their experience during the current experimental work, would like to recommend a detailed sustainability analysis on mortar samples containing ashes considering the treatment of ashes, their transport, and the manufacturing process.

## Figures and Tables

**Table 1:** Chemical compositions of calcined ashes.

Calcination Temperature	Compounds %	Duration			
		2 h	4 h	6 h	8 h
**WSA Calcination 300 °C**	SiO_2_	12.02	29.52	36.69	36.86
	Al_2_O_3_	1.417	0.888	0.737	0.661
	MgO	1.575	0.962	0.895	0.746
	CaO	1.017	0.811	0.777	0.674
	Na_2_O	0.553	0.458	0.364	0.256
	K_2_O	10.24	9.878	8.673	7.396
**WSA Calcination 600 °C**	SiO_2_	42.51	60.6	69.67	70.14
	Al_2_O_3_	1.644	0.737	0.586	0.51
	MgO	1.343	1.045	0.68	0.63
	CaO	0.857	0.72	0.594	0.457
	Na_2_O	0.701	0.634	0.377	0.283
	K_2_O	9.408	8.288	7.986	6.3
**WSA Calcination 800 °C**	SiO_2_	69.31	72.03	71.83	74.57
	Al_2_O_3_	1.682	0.869	0.737	0.586
	MgO	1.99	1.509	0.813	0.58
	CaO	0.925	0.788	0.605	0.537
	Na_2_O	1.011	0.701	0.445	0.377
	K_2_O	7.167	7.143	5.168	3.855
**RHA** **Calcination 300** **°C**	SiO_2_	47.92	47.619	52.432	63.62
	Al_2_O_3_	0.642	0.4535	0.3212	0.2834
	MgO	0.63	0.514	0.4809	0.3151
	CaO	1.508	1.0623	0.9024	0.3655
	Na_2_O	0.957	0.6605	0.5796	0.5662
	K_2_O	1.819	1.7828	1.7467	1.6503
**RHA** **Calcination 600** **°C**	SiO_2_	76.37	78.79	81.01	84.54
	Al_2_O_3_	1.493	0.926	0.737	0.529
	MgO	0.879	0.763	0.597	0.415
	CaO	2.033	1.165	1.074	0.537
	Na_2_O	0.634	0.62	0.553	0.526
	K_2_O	3.819	3.493	2.626	2.614
**RHA** **Calcination 800** **°C**	SiO_2_	80.86	91.9	92.26	92.37
	Al_2_O_3_	0.812	0	0.642	0.491
	MgO	0.614	0.464	0.448	0.431
	CaO	1.154	1.074	1.039	1.017
	Na_2_O	0.58	0.485	0.324	0.31
	K_2_O	3.24	2.325	2.204	2.108
**CDA** **Calcination 300** **°C**	SiO_2_	35.15	35.38	40.6	52.32
	Al_2_O_3_	3.458	2.154	2.154	2.967
	Fe_2_O_3_	2.902	2.145	1.859	1.887
	MgO	2.073	1.741	1.691	1.675
	CaO	5.46	3.004	2.993	2.833
	Na_2_O	1.348	1.146	0.957	0.876
	K_2_O	2.879	2.734	2.614	2.578
	P_2_O_5_	2.2	1.81	1.558	1.237
**CDA** **Calcination 600** **°C**	SiO_2_	49.2	61.97	74.25	75.41
	Al_2_O_3_	3.533	3.307	3.231	3.174
	Fe_2_O_3_	2.831	2.502	2.473	2.459
	MgO	2.106	1.94	1.807	1.807
	CaO	6.511	5.015	4.992	4.969
	Na_2_O	1.213	0.876	0.836	0.809
	K_2_O	2.674	2.59	2.554	2.518
	P_2_O_5_	1.994	1.581	1.329	0.962
**CDA** **Calcination 800** **°C**	SiO_2_	71.73	73.05	74.23	81.33
	Al_2_O_3_	3.571	3.363	3.231	2.003
	Fe_2_O_3_	3.074	2.788	1.944	3.431
	MgO	1.658	1.559	1.526	0.978
	CaO	6.877	6.408	6.134	6.054
	Na_2_O	1.2	0.849	0.795	0.782
	K_2_O	2.638	2.614	2.494	2.771
	P_2_O_5_	1.948	1.467	1.237	1.031

**Table 2:** Mix Details.

Mix ID	Cement	Sand	RHA	WSA	CDA	w/b
Control	1	2.75	-	-	-	0.49
RHA300-2H	0.80	2.75	0.20	-	-	0.49
RHA300-4H	0.80	2.75	0.20	-	-	0.49
RHA300-6H	0.80	2.75	0.20	-	-	0.49
RHA300-8H	0.80	2.75	0.20	-	-	0.49
RHA600-2H	0.80	2.75	0.20	-	-	0.49
RHA600-4H	0.80	2.75	0.20	-	-	0.49
RHA600-6H	0.80	2.75	0.20	-	-	0.49
RHA600-8H	0.80	2.75	0.20	-	-	0.49
RHA800-2H	0.80	2.75	0.20	-	-	0.49
RHA800-4H	0.80	2.75	0.20	-	-	0.49
RHA800-6H	0.80	2.75	0.20	-	-	0.49
RHA800-8H	0.80	2.75	0.20	-	-	0.49
WSA300-2H	0.80	2.75	-	0.20	-	0.49
WSA300-4H	0.80	2.75	-	0.20	-	0.49
WSA300-6H	0.80	2.75	-	0.20	-	0.49
WSA300-8H	0.80	2.75	-	0.20	-	0.49
WSA600-2H	0.80	2.75	-	0.20	-	0.49
WSA600-4H	0.80	2.75	-	0.20	-	0.49
WSA600-6H	0.80	2.75	-	0.20	-	0.49
WSA600-8H	0.80	2.75	-	0.20	-	0.49
WSA800-2H	0.80	2.75	-	0.20	-	0.49
WSA800-4H	0.80	2.75	-	0.20	-	0.49
WSA800-6H	0.80	2.75	-	0.20	-	0.49
WSA800-8H	0.80	2.75	-	0.20	-	0.49
CDA300-2H	0.80	2.75	-	-	0.20	0.49
CDA300-4H	0.80	2.75	-	-	0.20	0.49
CDA300-6H	0.80	2.75	-	-	0.20	0.49
CDA300-8H	0.80	2.75	-	-	0.20	0.49
CDA600-2H	0.80	2.75	-	-	0.20	0.49
CDA600-4H	0.80	2.75	-	-	0.20	0.49
CDA600-6H	0.80	2.75	-	-	0.20	0.49
CDA600-8H	0.80	2.75	-	-	0.20	0.49
CDA800-2H	0.80	2.75	-	-	0.20	0.49
CDA800-4H	0.80	2.75	-	-	0.20	0.49
CDA800-6H	0.80	2.75	-	-	0.20	0.49
CDA800-8H	0.80	2.75	-	-	0.20	0.49

## Data Availability

The data presented in this study are available on request from the corresponding author.
